# Reference intervals for serum osteocalcin concentrations in adult men and women from the study of health in Pomerania

**DOI:** 10.1186/1472-6823-13-11

**Published:** 2013-03-13

**Authors:** Anke Hannemann, Nele Friedrich, Christin Spielhagen, Rainer Rettig, Till Ittermann, Matthias Nauck, Henri Wallaschofski

**Affiliations:** 1 Institute of Clinical Chemistry and Laboratory Medicine, University Medicine Greifswald, Greifswald, Germany; 2 Institute of Physiology, University Medicine Greifswald, Greifswald-Karlsburg, Germany; 3 Institute for Community Medicine, University Medicine Greifswald, Greifswald, Germany; 4 Institute of Clinical Chemistry and Laboratory Medicine, University Medicine Greifswald, Greifswald, Germany

**Keywords:** Reference interval, Serum osteocalcin concentration, Healthy adult men and women

## Abstract

**Background:**

Osteocalcin (OC) is a bone-specific protein produced primarily by osteoblasts during bone formation. Besides its role in bone formation, osteocalcin may play a role in the regulation of energy metabolism and male fertility. To interpret serum OC data, reference intervals adapted to a specific laboratory method are needed.

**Methods:**

A healthy reference population was selected from the first follow-up of the Study of Health in Pomerania. Serum OC concentrations were measured with the IDS-iSYS N-Mid Osteocalcin assay on the IDS-iSYS Automated System (Immunodiagnostic Systems, Frankfurt am Main, Germany). The reference interval was defined as the central 95% range (2.5th-97.5th percentile). Age-specific reference intervals were calculated by quantile regression for 1107 men (25–79 years) and 545 premenopausal women (25–54 years). The reference interval for 498 postmenopausal women (50–79 years) was calculated irrespective of age.

**Results:**

Median (1st-3rd quartile) serum OC concentrations were 15.4 ng/mL (12.0-19.4 ng/mL) in men, 14.4 ng/mL (11.3-18.5 ng/mL) in premenopausal women, and 18.6 ng/mL (13.6-25.6 ng/mL) in postmenopausal women. Serum OC concentrations were highest in men and premenopausal women aged 25–29 years, were stable during midlife, and rose again after 65 years of age in men and at transition to menopause in women. Serum OC concentrations were lower in women taking oral contraceptives or who were under hormone replacement therapy after menopause and in subjects with diabetes mellitus or with body mass index < 18 or > 30 kg/m^2^ than in subjects without these conditions.

**Conclusions:**

We established sex-specific adult reference intervals for the serum OC concentration measured by the IDS-iSYS N-Mid Osteocalcin assay.

## Background

Bone is a dynamic tissue undergoing constant remodelling. In healthy adults maintenance of bone mass is achieved by coupling of bone formation and bone resorption processes [1]. These processes can be assessed by determination of bone turnover markers (BTMs) [2]. Although BTMs show promise for the determination of fracture risk, current evidence is insufficient to recommend the clinical application of BTMs in fracture risk prediction [3]. In addition to their potential role in fracture risk evaluation, BTMs indicate rapid changes in bone metabolism after initiation of antiresorptive or anabolic osteoporosis therapy [2,4,5]. It has been suggested that BTMs may aid selecting the optimal therapy and medication dose but also to monitor treatment compliance and efficacy in the individual patient [2,6].

There are several bone formation and resorption markers [2]. According to the 2012 European guidance for the diagnosis and management of osteoporosis in postmenopausal women [3], the most informative BTMs for osteoporosis monitoring are procollagen I N-terminal extension peptide and C-telopeptide breakdown products. Another BTM, characteristic for bone formation, is osteocalcin (OC). OC is a bone-specific protein produced primarily by osteoblasts during bone formation [2].

Besides its role in bone formation, several studies demonstrated a potential relevance of undercarboxylated OC in energy metabolism [7,8] and male fertility [9,10]. Also correlations between total OC, including undercarboxylated and carboxylated OC, and fasting glucose [11-13] or fasting insulin [12] were detected and it was demonstrated that OC levels are lower in diabetic than in healthy subjects [11,14].

Circulating OC concentrations vary according to age and sex [15-19]. Previous studies suggested that young adult men have higher levels than young women of the same age [20,21], as they have longer and wider bones and reach peak BMD later in life [17]. After peak BMD is reached, serum OC concentrations decline in men and women [15,17,19,22]. In middle-aged and elderly men beyond 50–60 years of age, serum OC concentrations become stable or increase slightly [15,16]. In premenopausal women between 35–45 years of age serum concentrations of OC and other BTMs are stable and it has been suggested, that female bone health is at its best at this age [19,22]. In women at transition to menopause, serum OC concentrations increase strongly [5]. Therefore, several investigators proposed to use the lower half of the OC reference interval for women between 35–45 years of age as the target for antiresorptive therapy in postmenopausal women [22-24].

There are several laboratory methods for OC measurement, which may differ in various aspects including the recognition of circulating OC fragments [25]. As the different OC assays may yield different results, it is of major importance to establish method-specific reference intervals to adequately interpret serum OC concentrations. Currently, most studies on reference intervals for serum OC concentrations focused on premenopausal women [19,22,23,26], while normative data for postmenopausal women [21] and men [16,21] are sparse. The aim of our study was to provide sex- and age-specific reference intervals for serum OC concentrations measured with the IDS-iSYS N-Mid Osteocalcin assay obtained in healthy adults aged 25–79 years.

## Methods

### The Study of Health in Pomerania (SHIP)

SHIP is a population-based cohort study in the northeast of Germany. Study design and sampling methods have been previously described [27]. In short, for the baseline study (SHIP-0) a representative sample of 7008 adults aged 20–79 years was drawn from the population living in the region of West Pomerania (n = 158,864). The study region comprised the three cities Greifswald, Stralsund and Anklam and the surrounding 29 communities. The baseline examinations were conducted in 4308 men and women between October 1997 and May 2001. Five years later, between March 2003 and July 2006, the first follow-up examination, designated as SHIP-1, was conducted with 3300 participants being re-examined. The present analyses are based on SHIP-1 data. Due to drop-out between baseline and follow-up examination, the SHIP-1 study population is not truly representative for the study region. All participants gave written informed consent. The study was reviewed by an external scientific review board and conformed to the principles of the Declaration of Helsinki as reflected by an *a priori* approval of the Ethics Committee of the Board of Physicians Mecklenburg-West Pomerania at the University of Greifswald.

### Instruments and measurements

Information on socio-demographic characteristics and medical histories was obtained by computer-aided personal interviews. Medication was classified using the Anatomical Therapeutic Chemical Classification System (ATC) code. Menopausal status was defined according to age and self-reported menstrual cycling. All women younger than 40 years of age and all women between 40 and 60 years of age who reported menstrual cycling were defined as premenopausal, all other women were defined as postmenopausal. It was not possible to define perimenopause as the respective information was not collected.

Non-fasting blood samples were taken from the cubital vein of participants in the supine position between 8.00 a.m. and 8.00 p.m. Serum aliquots were stored at −80°C. Serum OC concentrations were measured with the IDS-iSYS N-Mid Osteocalcin assay on the IDS-iSYS Multi-Discipline Automated Analyser (Immunodiagnostic Systems Limited, Frankfurt am Main, Germany) according to the instructions for use. This assay detects the intact OC polypeptide (amino acids 1–49) and the N-terminal-Mid OC fragment (amino acids 1–43). The measurement range of the assay was 2–200 ng/mL. The limits of blank and detection were 0.27 ng/mL. The limit of quantitation was 1.57 ng/mL. As recommended by the manufacturer, three levels of control material were measured in order to verify a decent working mode. During the course of the study, the coefficients of variation were 6.93% at low, 6.83% at medium, and 5.06% at high serum OC concentrations in the control material.

### Reference population

We selected a healthy reference population by excluding all subjects with missing data on serum OC concentration (n = 36), presence of or missing information on any of the following conditions: renal disease defined as estimated glomerular filtration rate (Cockcroft-Gault) <30 mL/min (n = 30), hyperparathyroidism defined as serum parathyroid hormone concentration >120 pg/mL (n = 24), hyperthyroidism defined according to local reference ranges [28] as serum thyroid-stimulating hormone concentration <0.25 mU/L and serum free thyroxine concentration >18.9 pmol/L (n = 71), or a self-reported history of cancer (n = 189), osteoporosis (n = 209), or liver disease (n = 41). In addition to these subjects, we excluded further participants with conditions known to affect bone metabolism including all pregnant women (n = 12), all subjects with serum 25-hydroxy vitamin D concentration <10 μg/L (n = 342), and all subjects (n = 85) who reported intake of any of the following medication: bisphosphonates (ATC M05BA, M05BB), selective estrogen receptor modulators (ATC G03XC), vitamin D (ATC A11CC), calcitonin (ATC H05BA), strontium ranelate (ATC M05BX03), parathyroid hormone (ATC H05AA), testosterone (ATC G03BA02, G03BA03), anticonvulsants (ATC N03A), heparin (ATC B01AB), steroids (ATC H02AB), calcium (ATC A12A), antiandrogens (ATC L02BB), or aromatase inhibitors (ATC L02BG). Moreover, due to the small number of subjects older than 79 years of age (n = 59 men and women) we also excluded those subjects. This resulted in a male reference population of 1107 subjects aged 25 to 79 years. In females we differentiated between pre- and postmenopausal women. As there were only few premenopausal women older than 54 years of age (n = 3) and few postmenopausal women younger than 50 years of age (n = 49) these women were also excluded from the analyses. This resulted in a reference population of 545 premenopausal women aged 25 to 54 years and 498 postmenopausal women aged 50 to 79 years.

### Statistical analyses

Continuous data are expressed as median (1st-3rd quartile) and nominal data are expressed as percentage. Group comparisons were performed using Kruskal-Wallis tests. One-way analysis of variance was used to test for significant effects of month of blood sampling, and significant effects of daytime of blood sampling on log-transformed mean serum OC concentrations. P-values <0.05 were considered statistically significant. To estimate reference limits nonparametric quantile regression was used, a method for estimating models for conditional quantile functions [29]. Unlike the linear regression approach, this method does not require that the data follow a Gaussian distribution. A transformation of the dependent variable was thus not necessary. To estimate the reference limits for serum OC concentrations as a function of age, we developed a polynomial model of the form f(age) = b + b_1_*age + b_2_*age^2^ + … + b^d^*age^d^. The highest degree d in the polynomial was determined by consecutively adding higher degree polynomials, until the added term was no longer statistically significant. The age-related changes of serum OC concentrations in men and premenopausal women were modeled by fitting a quadratic equation. The 2.5th and 97.5th percentiles of the OC distribution were estimated for each single year of age. Subsequently, mean values for five-year age groups were calculated. In postmenopausal women serum OC concentrations did not vary with age, thus an age-independent reference interval is reported. All statistical analyses were performed with SAS 9.1.3 (SAS Institute Inc., Cary, NC, USA).

## Results

In 1107 male subjects from the reference population the median serum OC concentration was 15.4 ng/mL (1st–3rd quartile: 12.0–19.4 ng/mL). In 545 premenopausal women the median serum OC concentration was 14.4 ng/mL (1st–3rd quartile: 11.3–18.5 ng/mL) and in 498 postmenopausal women the median serum OC concentration was 18.6 ng/mL (1st–3rd quartile: 13.6–25.6 ng/mL). Further characteristics of the reference population are presented in Table 1. Age-related changes in serum OC concentrations are displayed in Table 2.

**Table 1:** Characteristics of the reference population

**Characteristics**	**Men (n = 1107)**	**Women**	
		**Premenopausal (n = 545)**	**Postmenopausal (n = 498)**
Age [years]	53.0 (41.0 - 64.0)	39.0 (33.0 - 44.0)	62.0 (56.0 - 67.0)
BMI [kg/m^2^]	27.9 (25.5 - 30.7)	24.5 (22.1 - 28.1)	28.1 (25.0 - 32.1)
Diabetes mellitus [%]	10.1	0.92	13.1
Oral contraceptives (ATC G03A) [%]	-	23.1	-
Hormone replacement therapy (ATC G03C G03D G03F) [%]	-	-	9.6
25-hydroxy vitamin D [μg/L]	19.2 (14.6 - 25.1)	20.0 (14.5 - 28.3)	18.2 (13.7 - 23.8)
Parathyroid hormone [pg/mL]	33.7 (25.7 - 43.2)	28.4 (20.8 - 37.5)	36.3 (27.4 - 45.4)
Osteocalcin [ng/mL]	15.4 (12.0 - 19.4)	14.4 (11.3 - 18.5)	18.6 (13.6 - 25.6)

*BMI*, Body Mass Index.

Continuous variables are presented as median (1st - 3rd quartile), dichotomous variables are presented as proportions.

**Table 2:** Serum osteocalcin concentrations in five-year age groups

**Age group [years]**	**Men**		**Women**		
	**n**	**Serum OC concentration [ng/mL]**	**Menopausal status**	**n**	**Serum OC concentration [ng/mL]**
25-29	49	21.2 (16.5 - 25.0)	Pre-menopausal	71	17.2 (13.2 - 21.8)
30-34	79	18.9 (15.3 - 21.4)		96	15.5 (12.2 - 19.0)
35-39	113	17.4 (14.7 - 20.6)		125	14.5 (10.4 - 18.5)
40-44	113	16.9 (14.0 - 20.5)		118	13.4 (10.2 - 16.5)
45-49	123	14.5 (11.6 - 17.2)		87	12.9 (10.6 - 16.2)
50-54	127	13.5 (10.2 - 18.8)		48	14.2 (11.4 - 17.3)
			Post-menopausal	81	18.9 (12.5 - 27.3)
55-59	118	14.1 (11.2 - 17.8)		107	20.6 (14.3 - 28.6)
60-64	132	14.5 (11.4 - 18.1)		131	17.9 (13.5 - 22.8)
65-69	114	13.4 (10.8 - 17.2)		87	19.5 (12.9 - 26.3)
70-74	85	14.2 (10.4 - 18.5)		57	18.8 (13.7 - 28.0)
75-79	54	16.7 (12.3 - 20.0)		37	18.2 (14.4 - 25.4)

Values are presented as median (1st - 3rd quartile).

Throughout the calendar year there was little variation in serum OC concentrations. In the entire reference population the serum OC concentrations were lowest in March (median 15.0 ng/mL; 1st-3rd quartile: 11.9-19.1 ng/mL) and highest in December (median 16.9 ng/mL; 1st-3rd quartile: 13.5-22.3 ng/mL) (Additional file 1: Figure S1). Regarding daytime of blood sampling (Additional file 1: Figure S2), we observed no statistically significant variations in serum OC concentrations.

Premenopausal women using oral contraceptives (ATC G03A; n = 126) had significantly lower serum OC concentrations (median: 13.0 ng/mL; 1st-3rd quartile: 10.3-16.7 ng/mL) than premenopausal women not using oral contraceptives (median: 15.0 ng/mL; 1st-3rd quartile: 11.6-18.9 ng/mL; n = 419). Furthermore, postmenopausal women using hormone replacement therapy (ATC G03C G03D G03F; n = 48) had significantly lower serum OC concentrations (median: 14.9 ng/mL; 1st-3rd quartile: 11.9-19.9 ng/mL) than women not using hormone replacement therapy (median: 19.1 ng/mL; 1st-3rd quartile: 13.9-26.2 ng/mL; n = 450).

In addition, we found that men with diabetes mellitus or body mass index (BMI) <18 or >30 kg/m^2^ had significantly lower serum OC concentrations (median: 13.7 ng/mL; 1st-3rd quartile: 10.5-17.4, ng/mL; n = 391) than non-diabetic, normal weight men (median: 16.4 ng/mL; 1st-3rd quartile: 13.0-20.5, ng/mL; n = 716). Also pre- and postmenopausal women with diabetes mellitus or BMI <18 or >30 kg/m^2^ had significantly lower serum OC concentrations (premenopausal women median: 13.7 ng/mL; 1st-3rd quartile: 10.4-16.5 ng/mL; n = 105; postmenopausal women median: 16.7 ng/mL; 1st-3rd quartile: 11.9-22.3 ng/mL; n = 195) than non-diabetic, normal weight pre- and postmenopausal women (premenopausal women median: 14.6 ng/mL; 1st-3rd quartile: 11.5-18.8, ng/mL; n = 440; postmenopausal women median: 21.6 ng/mL; 1st-3rd quartile: 16.0-28.6, ng/mL; n = 255).

To account for the observed effects on the serum osteocalcin concentration, reference limits were determined (1.) for the entire reference population and (2.) after exclusion of women taking sex hormones for contraception or hormone replacement and subjects with diabetes mellitus or BMI <18 or >30 kg/m^2^.

**Figure 1:**
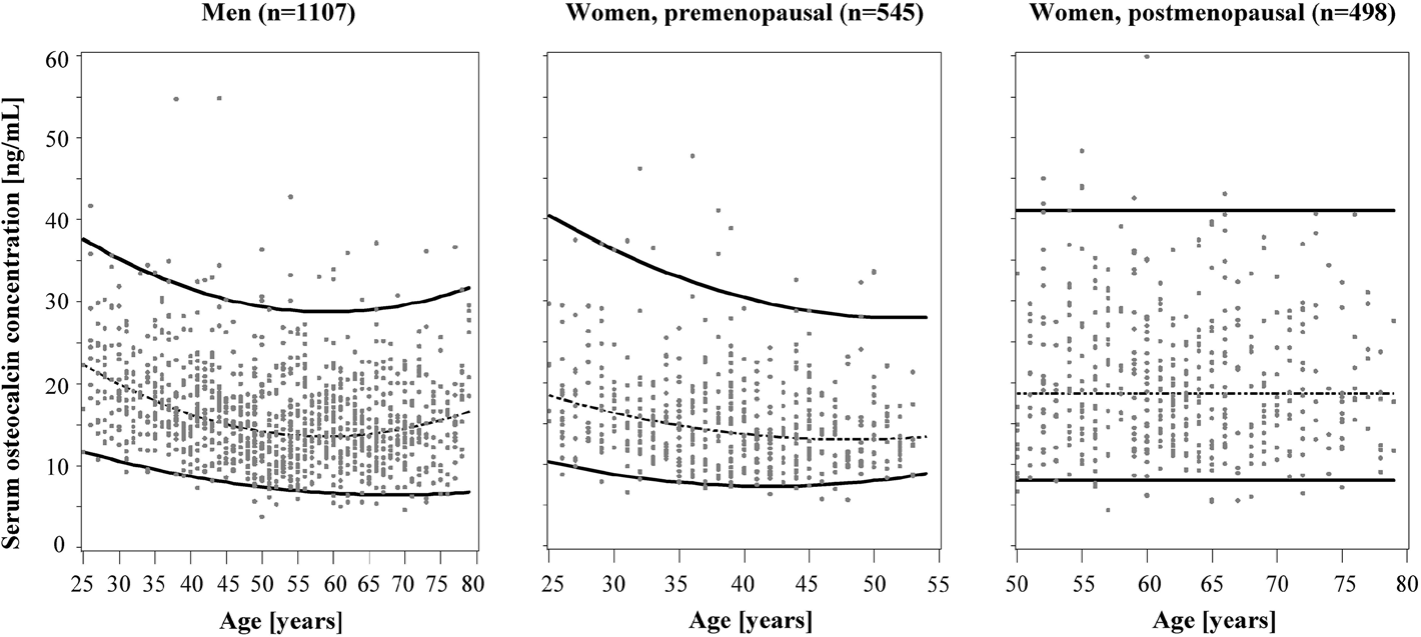
**Reference curves for serum osteocalcin concentrations in men as well as pre- and postmenopausal women.** Individual values (dots) and reference curves for the median (dashed lines) as well as the 2.5th and 97.5th percentiles (solid lines) are displayed. Reference limits for men and premenopausal women were determined by quantile regression. For postmenopausal women 2.5th and 97.5th percentiles were determined irrespective of age.

In men and premenopausal women reference limits were modeled by quantile regression. The models were fitted with parameters presented in Table 3. In postmenopausal women reference limits were determined irrespective of age. Reference intervals for five-year age groups are presented in Table 4. The respective reference curves are presented in Figure 1. In men aged 25–49 years upper and lower reference limits for serum OC concentrations decreased continuously with increasing age. In middle-aged and older men the upper and lower reference limits were relatively stable but increased slightly after age 65. In premenopausal women the upper and lower reference limits for serum OC concentrations decreased markedly between 25–34 years of age and remained stable after an age of 34 years. Previous studies [19,22] proposed that antiresorptive therapy should lower serum OC concentrations in postmenopausal women to the lower half of the reference interval of premenopausal women between 35–45 years of age. In our study, the reference interval for 35–44 year-old premenopausal women was 7.4-30.8 ng/mL, the lower half of the reference interval was (2.5th-50th percentile) 7.4-13.9 ng/mL.

**Table 3:** Parameter estimates with 95% confidence intervals (CI) for quantile regression models

**Model**	**Lower reference limit**		**Upper reference limit**	
	**(2.5th percentile)**		**(97.5th percentile)**	
	**Parameter estimate**	**(95% CI)**	**Parameter estimate**	**(95% CI)**
**Men**				
Intercept	19.4778	(15.7036; 26.1654)	55.3758	(42.6834; 76.1057)
Age	−0.3806	(−0.5235; -0.2307)	−0.8982	(−1.9454; -0.3549)
Age^2^	0.0028	(0.0012; 0.0052)	0.0076	(0.0029; 0.0181)
**Men, without diabetes mellitus and with BMI 18–30 kg/m**^**2**^				
Intercept	16.8043	(11.6050; 26.3003)	50.5885	(39.3315; 75.4833)
Age	−0.2382	(−0.5940; -0.0961)	−0.6898	(−2.2462; -0.2172)
Age^2^	0.0014	(−0.0001; 0.0048)	0.0058	(0.0010; 0.0158)
**Women, premenopausal**				
Intercept	25.825	(2.6944; 32.5959)	73.8844	(−37.2837; 310.5797)
Age	−0.8844	(−1.2162; 0.7307)	−1.7612	(−12.3023; 4.2155)
Age^2^	0.0106	(−0.0169; 0.0145)	0.0169	(−0.0551; 0.1337)
**Women, premenopausal without oral contraceptives, without diabetes mellitus, and with BMI 18–30 kg/m**^**2**^				
Intercept	46.8442	(1.4278; 53.8028)	62.0062	(−70.8969; 3609.508)
Age	−1.8757	(−2.2815; 0.0366)	−1.1167	(−66.4225; 6.5886)
Age^2^	0.0224	(−0.0006; 0.0279)	0.0104	(−0.0889; 0.8494)

*BMI*, Body Mass Index.

For postmenopausal women 2.5th and 97.5th percentiles were determined irrespective of age.

**Table 4:** Reference intervals (2.5th – 97.5th percentiles) for the serum osteocalcin concentration in five-year age groups

**Age group [years]**	**All men (N = 1107)**	**Men without diabetes mellitus and with BMI 18–30 kg/m**^**2 **^**(N = 716)**	**Women**	**All women (N = 545 premenopausal N = 498 postmenopausal)**	**Women without hormone intake, without diabetes mellitus and with BMI 18–30 kg/m**^**2 **^**(N = 440 premenopausal N = 255 postmenopausal)**
	**Reference interval [ng/mL]**	**Reference interval [ng/mL]**	**Menopausal status**	**Reference interval [ng/mL]**	**Reference interval [ng/mL]**
25-29	11.2 - 36.7	11.4 - 36.2	**Premenopausal**	9.7 - 38.7	12.6 - 39.5
30-34	10.2 - 34.4	10.6 - 34.5		8.4 - 34.8	9.8 - 37.0
35-39	9.2 - 32.5	9.9 - 33.1		7.6 - 31.9	8.1 - 35.0
40-44	8.4 - 31.0	9.2 - 31.9		7.3 - 29.7	7.6 - 33.5
45-49	7.7 - 29.9	8.6 - 31.1		7.6 - 28.4	8.2 - 32.6
50-54	7.2 - 29.2	8.1 - 30.5		8.4 - 28.0	9.8 - 32.1
			**Postmenopausal (all ages)**	8.0 - 40.9	10.4 - 43.8
55-59	6.8 - 28.8	7.7 - 30.3			
60-64	6.6 - 28.8	7.3 - 30.3			
65-69	6.5 - 29.2	7.0 - 30.6			
70-74	6.5 - 30.0	6.7 - 31.2			
75-80	6.7 - 31.2	6.5 - 32.1			

Reference limits for men and premenopausal women were determined by quantile regression and are displayed for five-year age groups. For postmenopausal women 2.5th and 97.5th percentiles were determined irrespective of age. Subjects with diabetes mellitus or BMI <18 or >30 kg/m^2^ and women using oral contraceptives or hormone replacement therapy had significantly lower serum OC concentrations than normal weight subjects without diabetes mellitus and women without intake of oral contraceptives or hormone replacement therapy. The exclusion of subjects with diabetes mellitus, BMI <18 or >30 kg/m^2,^ and women taking oral contraceptives or hormone replacement therapy shifted the distribution of serum osteocalcin concentrations and the reference intervals to higher values.

**Figure 2:**
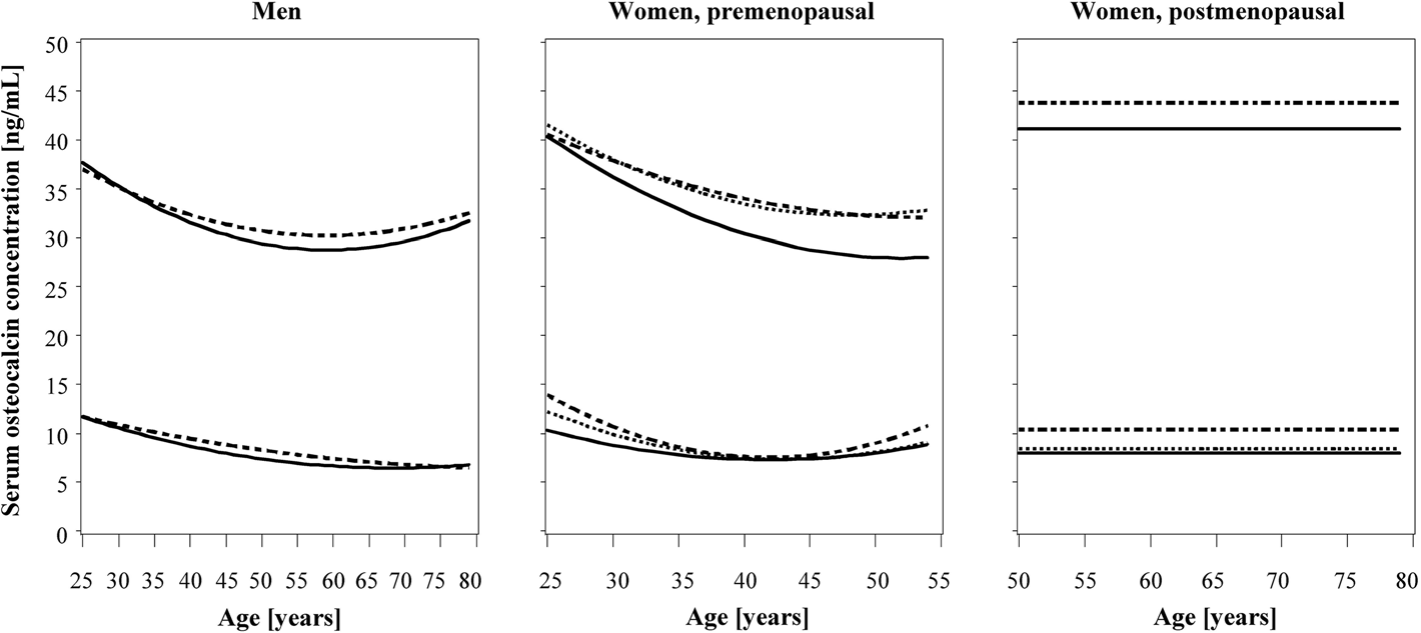
**Reference curves for serum osteocalcin concentrations from the whole reference population and from two subpopulations.** Reference curves include (1.) all subjects from the reference population (solid lines), (2.) premenopausal women not taking oral contraceptives or postmenopausal women without hormone replacement therapy (dotted lines) and (3.) all subjects without diabetes mellitus or with BMI <18 or >30 kg/m^2^ (dashed lines). Reference limits for men and premenopausal women were determined by quantile regression. For postmenopausal women 2.5th and 97.5th percentiles were determined irrespective of age.

The reference limits in subjects not taking oral contraceptives or hormone replacement therapy, without diabetes mellitus and with BMI between 18–30 kg/m^2^ (716 men, 329 pre- and 255 postmenopausal women) were higher than the reference limits obtained in the whole reference population (Table 4). This is particularly true for the upper reference limits, whereas the lower reference limits were less affected (Figure 2). In premenopausal women not taking oral contraceptives, without diabetes mellitus and with BMI between 18–30 kg/m^2^ the reference interval for 35–44 year-old women was 7.8-34.2 ng/mL, and the lower half of the reference interval was 7.8-14.4 ng/mL.

## Discussion

We established reference intervals for serum OC concentrations measured with the IDS-iSYS N-Mid Osteocalcin assay in a large cohort of subjects with European ancestry. We observed age- and sex-specific differences in serum OC concentrations in healthy adults. Next to age and sex, the normative data was influenced by intake of oral contraceptives or hormone replacement therapy as well as by altered anthropometry (BMI <18 or >30 kg/m^2^) and diabetes mellitus.

The estimated reference intervals for men, as well as pre- and postmenopausal women follow previously described age-related patterns [5,17,19,22]. In premenopausal women reference limits were highest in the youngest age group of 25–29 years. In premenopausal women older than 34 years of age, reference intervals were relatively stable. The relatively high serum osteocalcin concentrations in premenopausal women below 30 years of age may indicate that peak bone mass is not yet reached at this age, while the stable and low serum osteocalcin concentrations in premenopausal women aged 35 or older may indicate that these women have reached skeletal maturity and a phase of stable bone turnover [22]. Afterwards, at transition to menopause, the loss of estrogen results in a disequilibrium between bone formation and bone resorption processes [30,31]. During this period, serum OC concentrations rise considerably, by about 50-150% [5]. Our data are in line with these results: median serum OC concentrations and serum OC reference limits increased strongly in postmenopausal women compared to premenopausal women. Especially the upper reference limit for postmenopausal women (40.9 ng/mL) was markedly higher than the upper reference limit for premenopausal women aged 50–54 years (28.0 ng/mL). Male reference limits decreased between 25 to 49 years of age, were stable between 50 to 65 years of age, and increased minimally in the late 6th and 7th decade of life. The increase in serum OC reference limits in elderly men was, however, not comparable to the increase observed in females during transition to menopause.

In addition to the effects of age and sex, there may be other factors influencing serum OC concentrations such as the use of oral contraceptives [23,32,33], hormone replacement therapy [18,34], diabetes mellitus [35,36], and BMI [18,23]. Furthermore, serum OC concentrations were reported to be affected by calcium intake [19,37] and to be subject to circadian [38,39], or seasonal variations [18,40,41]. The potential effects of these factors on serum OC concentrations are controversial. While we did not observe circadian or seasonal variations of serum OC concentrations in our study population, our data confirm the influence of oral contraceptives, hormone therapy, diabetes mellitus, and BMI on serum OC concentrations. Overall, the examined factors had stronger impacts on the upper than on the lower reference limits. After exclusion of all subjects using oral contraceptives or hormone replacement therapy, with diabetes or BMI < 18 or >30 kg/m^2^, the upper reference limits increased by maximally 4.2 ng/mL in women and by maximally 1.5 ng/mL in men while the lower reference limits increased by maximally 2.9 ng/mL in women and by maximally 0.9 ng/mL in men.

In general, female osteoporosis has been more intensively investigated than male osteoporosis, probably because the prevalence of osteoporotic fractures is much higher in women than in men [42]. The focus on female osteoporosis is reflected in the larger number of studies reporting female [19,21-23,26] than male [21] reference intervals for serum OC concentrations. For premenopausal women previous studies report serum OC reference intervals based on data from 153 women between 35–45 years of age from the U.K. (7.0-28.3 ng/mL) [22], 82 women between 46–50 years of age from Italy (1.91-4.87 ng/mL) [23], 765 women between 35–45 years of age from Saudi Arabia (2.47 – 16.78 ng/mL) [19], and 475 women between 30–44 years of age from Japan (2.0-7.6 ng/mL) [26]. In the study from the U.K. [22] serum OC concentrations were measured with an enzyme-linked immunosorbent assay (ELISA) from Nordic Bioscience Diagnostics. In the Italian [23] and Saudi Arabian studies [19] immunoassays from Roche Diagnostics were used, and in the Japanese study [26] an immunoradiometric assay from Mitsubishi Kagaku Iatron Inc. was used. Our reference interval for 35–44 year-old premenopausal women (7.4-30.8 ng/mL) is in good agreement with that reported for premenopausal women from the U.K. [22], while those from Italy [23], Saudi Arabia [19], and Japan are considerably lower [26]. It is well known that different OC assays may yield different results [25]. The main reasons for this include differences in recognition of circulating OC fragments and differences in cross-reactivity to other molecules [25]. Next to differences in laboratory methods also sample handling influences the serum OC concentration, as it is sensitive to freeze-thaw cycles and haemolysis [25]. In how far the laboratory methods and the sample handling contributed to the differences in the serum OC reference intervals reported above cannot be quantified in present study. Yet, the differences between the laboratory methods may not be large, as the assays used in the studies from the U.K. [22], Italy [23], and Saudi Arabia [19] detect the intact OC polypeptide and the N-terminal-Mid OC fragment, as does the assay used in the present study.

Serum OC concentrations are further affected by ethnicity. In a study conducted in 2313 pre- or early perimenopausal Caucasian, African American, Chinese, and Japanese women serum OC concentrations were higher in Caucasian women than in all other ethnic groups [43]. Ethnic differences in serum OC concentrations may thus explain the lower reference intervals obtained in women from Iran (30–40 years: 3.46-19.95 ng/mL; 40–50 years: 2.88-26.30) [21] compared to those obtained in women from the U.K. [22], although serum osteocalcin concentrations were measured with the same assay. The Iranian study [21] also reported reference intervals for men. The reference limits for men aged 30 years and older were lower than those obtained in our study, probably due to ethnic differences.

In the current European guidance for the diagnosis and management of osteoporosis in postmenopausal women [3] P1NP, but not OC, is recommended for the laboratory assessment of bone formation. Nevertheless, normative, method-specific OC data can be applied to assess individual OC levels. Moreover, normative OC data may be useful to investigate the relation between OC and energy metabolism [44,45], as OC has been suggested to be a biomarker for insulin resistance [44].

Our study has several strengths and limitations. Strengths include the large sample size of extensively characterized study participants. Moreover, all laboratory measurements were performed in a central laboratory by trained personnel according to the manufacturers’ recommendations. A major limitation of our study is the lack of BMD measurement in our study participants. Thus, we could not verify whether subjects who did not report osteoporosis were truly healthy or were yet unaware of an existing osteoporosis. This may have led to the inclusion of subjects with unknown osteoporosis in the reference population and to inaccuracies in the estimated reference intervals. Another limitation relates to the definition of menopausal status, which was based on age and self-reported menstrual cycling. As perimenopause could not be defined, a misclassification of the respective women as pre- or postmenopausal may have occurred. This in turn could have biased pre- and postmenopausal reference intervals. Yet, the consistency of our results with those reported for premenopausal women from the U.K. [22] suggests that our study accurately reflects the reference ranges for serum OC concentrations in healthy premenopausal women. Moreover, our reference ranges are method-sensitive and may not be applicable to other methods of OC measurement. In addition, our sample is restricted to Caucasian subjects. It may therefore not be appropriate to apply our reference ranges to other ethnicities.

## Conclusions

We presented reference intervals for serum OC concentrations measured by the IDS-iSYS N-Mid Osteocalcin assay for men as well as for pre- and postmenopausal women from Northeast Germany. In addition to age and sex-related differences, we further detected an influence of oral contraceptives, hormone replacement therapy, diabetes mellitus, and BMI on serum OC concentrations in healthy Caucasian subjects.

## Competing interest

The authors declare that there is no conflict of interest that could be perceived as prejudicing the impartiality of the research reported.

## Author contributions

Study design, data analysis, and data interpretation: AH, NF, CS, RR, TI, MN and HW. Drafting manuscript and revising manuscript content: AH, NF, CS, RR, TI, MN and HW. Approving final version of manuscript: AH, NF, CS, RR, TI, MN and HW.

## Funding

This work was funded by grants from the German Federal Ministry of Education and Research (BMBF, Grants 01ZZ0403, 01ZZ0103, 01GI0883), the Ministry for Education, Research and Cultural Affairs as well as the Ministry of Social Affairs of the Federal State of Mecklenburg-West Pomerania. This work is also part of the research project Greifswald Approach to Individualized Medicine (GANI_MED). The GANI_MED consortium is funded by the Federal Ministry of Education and Research and the Ministry of Cultural Affairs of the Federal State of Mecklenburg – West Pomerania (03IS2061A). Furthermore, we received an independent research grant for determination of serum samples from Immunodiagnostic Systems.

## Pre-publication history

The pre-publication history for this paper can be accessed here:

http://www.biomedcentral.com/1472-6823/13/11/prepub

## Supplementary Material

Additional file 1: Figure 1 Box-plots for seasonal variation in serum osteocalcin concentrations in 2150 subjects from the reference population. The width of the plots is proportional to the number of blood samples obtained in each month. Five extreme observations have been omitted. **Figure 2.** Box-plots for variation of serum osteocalcin concentrations in 2150 subjects from the reference population by time of day. The width of the plots is proportional to the number of blood samples obtained during each hour of the day. Five extreme observations have been omitted. Click here for file
