# Supplementary material for: Reference intervals for serum osteocalcin concentrations in adult men and women from the study of health in Pomerania
Source: BMC Endocr Disord. 2013 Mar 13;13:11. doi: 10.1186/1472-6823-13-11 (PMC3606466; doi:10.1186/1472-6823-13-11)
Supplement: Additional file 1: Figure 1 — Box-plots for seasonal variation in serum osteocalcin concentrations in 2150 subjects from the reference population. The width of the plots is proportional to the number of blood samples obtained in each month. Five extreme observations have been omitted. Figure 2. Box-plots for variation of serum osteocalcin concentrations in 2150 subjects from the reference population by time of day. The width of the plots is proportional to the number of blood samples obtained during each hour of the day. Five extreme observations have been omitted. [file 1472-6823-13-11-S1.doc]

**Additional Material**

**Additional Figure 1.** Box-plots for seasonal variation in serum osteocalcin concentrations in 2150 subjects from the reference population. The width of the plots is proportional to the number of blood samples obtained in each month. Five extreme observations have been omitted.

**Additional Figure 2.** Box-plots for variation of serum osteocalcin concentrations in 2150 subjects from the reference population by time of day. The width of the plots is proportional to the number of blood samples obtained during each hour of the day. Five extreme observations have been omitted.


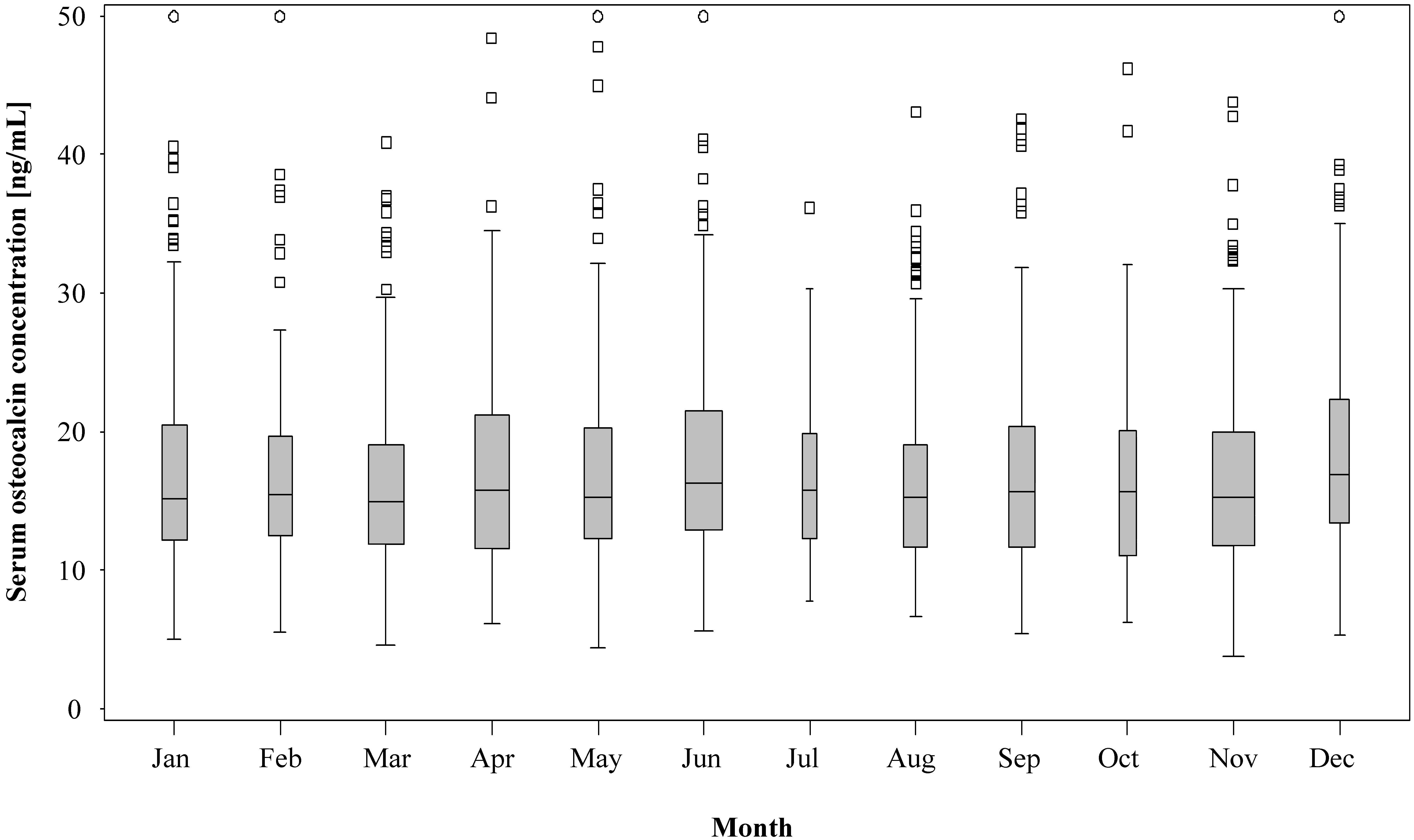


**Additional Figure 1.**


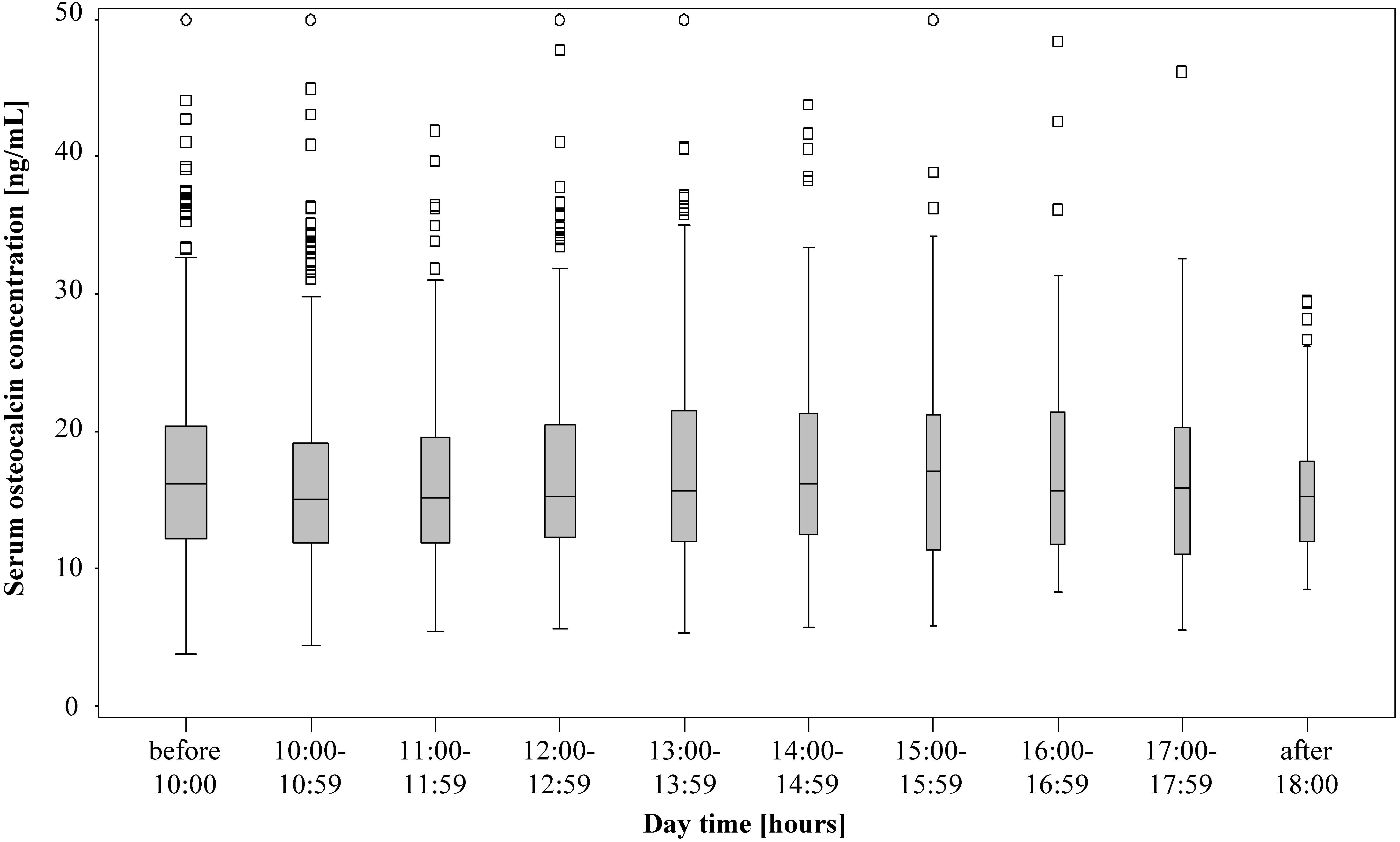


**Additional Figure 2.**
